# Cough in the Elderly During the COVID-19 Pandemic

**DOI:** 10.1007/s00408-022-00525-2

**Published:** 2022-03-17

**Authors:** Johanna Tuulikki Kaulamo, Anne Marika Lätti, Heikki Olavi Koskela

**Affiliations:** 1 Unit for Medicine and Clinical Research, Pulmonary Division, Kuopio University Hospital, Kuopio, Finland; 2 Faculty of Health Sciences, Institute of Clinical Sciences, School of Medicine, University of Eastern Finland, Yliopistonranta 1, 70210 Kuopio, Finland; 3 Mehiläinen Terveyspalvelut Oy, Health Care Services for Prisoners, Kuopio, Finland

**Keywords:** Chronic cough, Cough prevalence, Asthma, Upper airway cough syndrome, GERD cough, OSA cough

## Abstract

**Introduction:**

The prevalence of chronic cough increases with age. However, data on the prevalence and background disorders of cough subtypes in the elderly are scarce. The objective of this study was to identify the point prevalence and risk factors of acute, subacute, and chronic cough in an elderly community-based population.

**Methods:**

This was a cross-sectional email survey amongst 26,205 members of the Finnish Pensioners’ Federation during the COVID-19 pandemic in spring 2021.

**Results:**

The response rate was 23.6% (6189). 5983 subjects aged at least 64 years were included in the analyses (mean 72.6 years, 66.3% female). The point prevalence of daily acute, subacute, and chronic cough were 1.4%, 0.7%, and 9.6%, respectively. Only 0.4% of the subjects had a COVID-19 infection. In the multivariate analyses, chronic rhinosinusitis, and obstructive sleep apnoea were common risk factors for all cough subtypes. Chronic cough had several risk factors; Bronchiectasis (OR 5.79 (CI95% 2.70–12.41)), current asthma (2.67 (2.02–3.54)), chronic rhinosinusitis (2.51 (1.94–3.24)), somatic symptom score (1.13 per symptom (1.07–1.19)), family history of chronic cough (1.88 (1.54–2.30)), gastro-oesophageal reflux disease (1.86 (1.50–2.32)), advanced age (1.20 per decade (1.02–1.40)), chronic obstructive pulmonary disease (1.74 (0.99–3.05)), dog ownership (1.42 (1.07–1.89)), and obstructive sleep apnoea (1.41 (1.16–1.73)).

**Conclusion:**

Acute and subacute cough, as well as previous COVID-19 infection, were uncommon in this Finnish elderly population. The prevalence of chronic cough was higher than that previously found in younger adults. Chronic cough is a multifactorial disorder in the elderly.

**Supplementary Information:**

The online version contains supplementary material available at 10.1007/s00408-022-00525-2.

## Introduction

Cough is the most common reason to seek medical help worldwide [1]. Cough is categorised as acute (< 3 weeks), subacute (3–8 weeks) or chronic (> 8 weeks) by duration [2]. Chronic cough is often refractory to treatment, causes impairment in the health-related quality of life and leads to repetitive doctors’ visits [3, 4]. Thus, cough causes a high socioeconomic burden.

The point prevalence of daily acute, subacute, and chronic cough in Finnish working age adults are 5.4%, 3.4%, and 7.2%, respectively [5]. The prevalence of chronic cough increases with age [5–10]. To our knowledge, there are no studies focusing on the point prevalence and risk factors of cough subtypes specifically in the elderly.

Acute cough is usually a manifestation of an acute respiratory infection (ARI) [2]. Subacute cough can also spark from an ARI; however, cough prolongation may involve background disorders, like allergy, chronic rhinosinusitis, and asthma [5]. The management of chronic cough still relies on the recognition and treatment of the background disorders [2, 11]. The aetiology of chronic cough may be different in the elderly than in younger adults [12–14], but the subject remains under researched. As the population is getting older globally, better knowledge about the underlying reasons of chronic cough in the elderly is needed.

We have previously performed a community-based study about prevalence and risk factors of cough in Finnish working age population [5]. In 2021, the survey was repeated in an elderly population for comparison. The aim of this study was to investigate the point prevalence and risk factors of acute, subacute, and chronic cough in subjects aged at least 64 years in spring 2021, which was during the COVID-19 pandemic.

## Materials and Methods

### Population

This was an observational, cross-sectional study conducted via email amongst the members of the Finnish Pensioners’ Federation. It is the largest pensioner organisation in Finland with about 120,000 members. The 26,205 members (mean age 72.7 years, 63.5% female), who had an email address, were sent an invitation to participate along with information about the study. The questionnaires were sent in 5th April 2021 and one reminder email was sent 2 weeks later. The subjects were asked to respond by 30th April 2021. The responses were directly recorded in an electronic datasheet. A replied questionnaire was considered as an informed consent. The study was approved by the Ethics Committee of Kuopio University Hospital (289/2015). Permission to conduct the study was obtained from the Finnish Pensioners’ Federation. Patients were not involved in the design or conduct of this study.

### The Questionnaire

The questionnaire consisted of 62 questions about date of birth, social background, lifestyle, general health, doctors’ diagnoses and visits, and medication. Validated symptom questionnaires for current asthma [15], chronic rhinosinusitis [16], gastro-oesophageal reflux disease (GORD) [17], and obstructive sleep apnoea (OSA) [18, 19] were included in the questionnaire. Respondents with current cough answered 24 additional cough-related questions. The same questionnaire was used in our previous study in working age population [5]. For this study, 6 new questions were added about OSA, COVID-19 infection, and recurrence of cough. An English version of the questionnaire is provided as a Supplementary File.

### Definitions

Current cough was defined as presence of cough within 2 weeks. Current cough with a bout frequency of at least once a day was used as the primary outcome in the risk factor analyses. Acute cough was defined as cough that has lasted for less than 3 weeks, subacute cough for 3–8 weeks, and chronic cough for over 8 weeks. Any daily cough was defined as current daily cough of any duration. Current asthma was defined as presence of wheezing within 12 months, presence of wheezing without an ARI, and dyspnoea during wheezing [15]. Chronic rhinosinusitis was present if there was either nasal blockage or discharge (anterior or posterior nasal drip) and either reduction/loss of smell or facial pain/pressure for at least 3 months in the past year [16]. GORD was defined as presence of heartburn or regurgitation at least once a week in the last 3 months [17]. OSA was defined as presence of 2 or more of the following features: Loud snoring, daytime tiredness, observed apnoeas, and arterial hypertension (STOP-questionnaire [18, 19]). Somatic symptom score was defined as the sum (0–15) of experienced symptoms during the last month, excluding respiratory symptoms [5]. Family history of chronic cough was defined as cough lasting for over 8 weeks in parents or siblings. COVID-19 infection was defined as a self-reported laboratory-confirmed diagnosis of the infection. Allergy was defined as self-reported allergy to animals, pollens, or food. Connective tissue disease was defined as doctor’s diagnosis of rheumatoid arthritis or other inflammatory disease of connective tissue. Any other illness mentioned, such as chronic obstructive pulmonary disease (COPD) or bronchiectasis, was defined as self-reported doctor’s diagnosis of the disease in question.

### Statistical Analysis

Categorical data are expressed as percentages and continuous data as means and standard deviations (SD). The bivariate associations were analysed with *X*^2^ test and Mann–Whitney *U* test. The bivariate associations between the cough subtypes and the following characteristics were investigated: age, gender, body mass index (BMI), smoking, family history of chronic cough, COVID-19 infection, moisture damage exposure, pet ownership, allergy, chronic rhinosinusitis, current asthma, COPD, bronchiectasis, pulmonary fibrosis, sarcoidosis, tuberculosis, GORD, OSA, somatic symptom score, diabetes, coronary artery disease, medications for arterial hypertension and diabetes, connective tissue diseases, hypothyroidism, and Parkinson’s disease. Only the variables with plausible biological association with cough were included in the multivariate analyses [20]. The multivariate analyses were conducted using binary logistic regression with backward-directed stepwise exclusion. Statistical significance was accepted for *p*-value less than 0.05. Suggestive associations with *p*-values less than 0.1 are also presented. All analyses were conducted using SPSS v. 27.

## Results

**Fig. 1:**
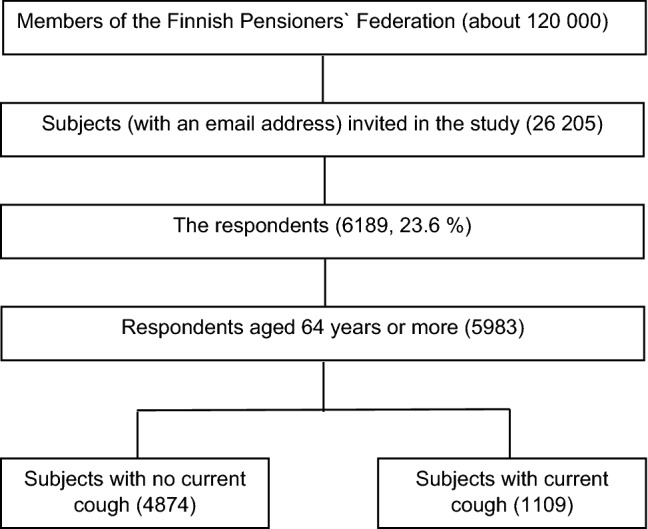
Flowchart of the study population

The response rate was 23.6% (6189 respondents, mean age 72.2 (5.5) years, 66.4% female, (Fig. 1)). The proportion of missing values was less than 2.5%, except for the 3 OSA-related symptom questions (3.1–3.7%). Due to age less than 64 years, 206 respondents were excluded from the analyses. Of the remaining 5983 respondents, the mean age was 72.6 years (SD 5.1, range 64–94), 66.3% were female, 1.7% currently daily smokers, and 35.2% ever-smokers. The mean BMI was 27.4 (4.6).

**Fig. 2:**
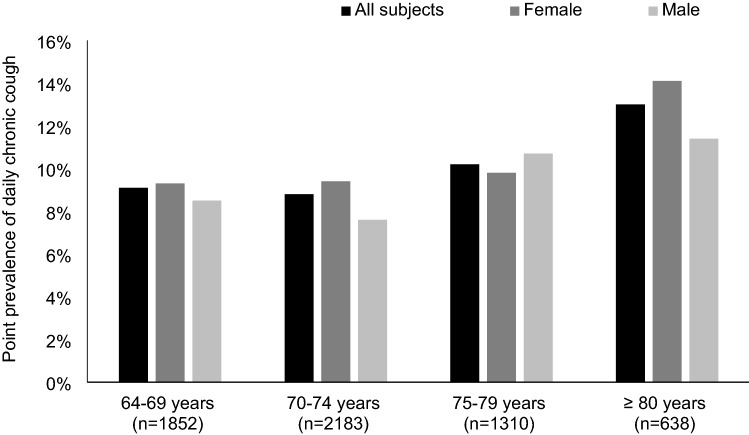
The point prevalence of daily chronic cough by age group and gender

The point prevalence of any daily cough was 11.8%. The point prevalence of daily acute, subacute, and chronic cough were 1.4%, 0.7%, and 9.6%, respectively (Table 1). The prevalence of chronic cough increased with age (Fig. 2).

**Table 1:** Point prevalence of cough with different bout frequencies

Cough bout frequency	Acute cough, %	Subacute cough, %	Chronic cough, %	Any cough, %
Any	3.5	1.2	13.5	18.5
Once a week or more often	2.5	1.1	12.8	16.6
2–3 days a week or more often	2.2	1.0	11.9	15.2
4–6 days a week or more often	1.6	0.8	10.9	13.4
Once a day or more often	1.4	0.7	9.6	11.8

The results of the bivariate analyses of cough background factors are presented in Table 2. ARI at onset of cough was present in 41.2% of subjects with acute cough, 27.5% with subacute cough, and 13.7% with chronic cough. COVID-19 infection had been diagnosed in 0.4% of the respondents.

**Table 2:** Characteristics of the subjects and bivariate associations of each cough subtype compared to subjects with no current cough

Characteristic	No current cough (*n* = 4874)	Acute daily cough (*n* = 85)	Subacute daily cough (*n* = 40)	Chronic daily cough (*n* = 577)
Age, years	72.5 (5.0)	72.8 (5.5)	71.6 (5.1)	73.2 (5.2)*
Female gender, %	66.0	70.6	75.0	68.1
Body mass index, kg/m^2^	27.3 (4.5)	27.2 (3.8)	28.8 (5.5)	27.8 (4.7)**
Current smoker, %	1.7	2.4	2.6	2.4
Ever-smoker, %	34.5	34.1	35.0	39.2*
Family history of chronic cough, %	21.9	25.0	47.5***	42.9***
Acute respiratory infection at onset of cough, %	NA	41.2	27.5	13.7
COVID-19 infection, %	0.3	2.4**	2.5*	0.7
Moisture damage exposure, %	1.5	1.2	5.0	1.4
Pet ownership, %	17.8	28.2*	25.0	19.4
Allergy, %	7.9	17.6**	22.5***	15.9***
Chronic rhinosinusitis, %	7.2	17.6***	25.0***	22.7***
Current asthma, %	5.0	14.3***	17.5***	20.9***
Chronic obstructive pulmonary disease, %	1.3	2.4	0.0	4.9***
Bronchiectasis, %	0.4	0.0	2.5*	3.1***
Pulmonary fibrosis, %	0.3	4.7***	0.0	1.4***
Sarcoidosis, %	0.4	1.2	0.0	0.5
Tuberculosis, %	0.0	1.2***	0.0	0.0
Gastro-oesophageal reflux disease, %	15.0	21.2	20.0	32.4***
Obstructive sleep apnoea, %	28.3	47.5***	50.0**	47.0***
Somatic symptom score	1.9 (1.7)	2.9 (2.2)***	2.5 (1.6)**	2.9 (2.0)***
Diabetes, %	11.9	9.4	22.5*	13.9
Diabetes medication, %	11.7	8.2	20.0	12.5
Coronary artery disease, %	5.6	10.6*	10.0	8.1*
Antihypertensive medication, %	50.3	61.2*	65.0	56.0*
Connective tissue disease, %	4.8	8.2	10.0	6.2
Hypothyroidism, %	13.4	17.6	15.0	16.5*
Parkinson’s disease, %	0.3	0.0	0.0	0.3

*NA* not applicable

*p < 0.05, **p < 0.01, ***p < 0.001

The results of the multivariate analyses of any daily cough and the cough subtypes are presented in Tables 3 and 4. Bronchiectasis and previous COVID-19 infection had the strongest associations with any daily cough. Chronic rhinosinusitis and OSA were associated with all cough subtypes. Allergy was associated with the shorter cough subtypes. Family history of chronic cough was associated with the longer cough subtypes. There were 10 risk factors of chronic cough with bronchiectasis, current asthma, and chronic rhinosinusitis being the strongest. Several of the risk factors were specific for chronic cough.

**Table 3:** Characteristics associated with any daily cough (*n* = 702) in the multivariate analysis, in comparison to subjects with no current cough (*n* = 4874)

Characteristic	Adjusted OR and 95% CI
Bronchiectasis	4.84 (2.30–10.17)***
COVID-19 infection	2.91 (1.07–7.91)*
Current asthma^a^	2.58 (1.99–3.35)***
Chronic rhinosinusitis^a^	2.38 (1.87–3.03)***
Pulmonary fibrosis	2.36 (1.02–5.49)*
Family history of chronic cough	1.79 (1.48–2.16)***
Gastro-oesophageal reflux disease^a^	1.66 (1.35–2.03)***
Allergy	1.44 (1.11–1.88)**
Obstructive sleep apnoea^a^	1.43 (1.19–1.72)***
Dog ownership	1.39 (1.06–1.81)*
Age^b^	1.17 (1.01–1.34)*
Somatic symptom score^c^	1.13 (1.08–1.18)***

ORs are presented if *p* < 0.10

*p < 0.05, **p < 0.01, ***p < 0.001

^a^Defined by a validated questionnaire [15–19]

^b^OR expressed per decade

^c^OR expressed per symptom

**Table 4:** Adjusted ORs and 95% confidence intervals for characteristics in all cough subtypes with daily bout frequency, compared to subjects with no current cough (*n* = 4874)

Characteristic	Acute cough (*n* = 85)	Subacute cough (*n* = 40)	Chronic cough (*n* = 577)
Chronic rhinosinusitis^a^	2.21 (1.21–4.04)*	3.20 (1.52–6.74)**	2.51 (1.94–3.24)***
Obstructive sleep apnoea^a^	1.62 (1.02–2.60)*	1.81 (0.96–3.42)	1.41 (1.16–1.73)***
Family history of chronic cough	NS	2.61 (1.38–4.93)**	1.88 (1.54–2.30)***
Allergy	2.19 (1.21–3.95)**	2.60 (1.21–5.61)*	NS
Current asthma^a^	NS	NS	2.67 (2.02–3.54)***
Cat ownership	2.31 (1.25–4.26)**	NS	NS
Dog ownership	NS	NS	1.42 (1.07–1.89)*
Somatic symptom score^b^	1.16 (1.04–1.30)**	NS	1.13 (1.07–1.19)***
Bronchiectasis	NS	NS	5.79 (2.70–12.41)***
Chronic obstructive pulmonary disease	NS	NS	1.74 (0.99–3.05)
Gastro-oesophageal reflux disease^a^	NS	NS	1.86 (1.50–2.32)***
Age^c^	NS	NS	1.20 (1.02–1.40)*

ORs are presented if *p* < 0.10

*NS* not significant (*p* > 0.10)

*p < 0.05, **p < 0.01, ***p < 0.001

^a^Defined by a validated questionnaire [15–19]

^b^OR expressed per symptom

^c^OR expressed per decade

## Discussion

In this community-based Finnish elderly population of 5983 subjects, we investigated the point prevalence and risk factors of all cough subtypes during the COVID-19 pandemic. The results showed that very few subjects had been infected with SARS-CoV-2. Furthermore, the prevalence of acute and subacute coughs were low in the elderly (1.4% and 0.7%, respectively) in comparison with Finnish working age adults in 2017 (5.4% and 3.4%, respectively) [5]. Consequently, the prevalence of any daily cough was also lower than in younger adults (11.8% *vs*. 16.1%). A possible reason for the discrepancy is the use of personal protective and social measures which were highly recommended during the pandemic, especially for the elderly. As a result, prevalence of the short, ARI-associated cough subtypes may have decreased in this population. According to previous reports, also the prevalence of ARI-induced disorders, such as exacerbations of asthma, COPD, and bronchiectasis, decreases during lockdown [21–23]. These disorders are typically characterised by cough, and the decrease of their prevalence may also contribute to the rarity of acute and subacute cough in the present population. Thus, personal protective and social measures may be effective in cutting down the burden of acute and subacute cough.

The point prevalence of daily chronic cough in this Finnish elderly population was higher than in Finnish working age adults (9.6% *vs.* 7.2%) [5]. The prevalence of chronic cough with any bout frequency (13.5%) was also higher than the prevalence estimate in European adults (12.7%) [24]. A previous Korean study included an elderly subgroup, in which the point prevalence of chronic cough was significantly lower (about 5.5%) than in the present study [7]. These results align well with earlier epidemiological studies regarding prevalence differences according to age and region. However, inconsistent definitions of chronic cough and reporting of prevalence (period *vs.* point prevalence) somewhat hinder global comparison. Considering the understanding of the aetiology of chronic cough and the present results, it seems that chronic cough and its burden remain unaffected by personal protective and social measures against ARIs.

In the multivariate analysis of any daily cough, a higher number of risk factors were recognised in the elderly than in working age adults [5], corroborating that cough becomes a more multifactorial entity toward the higher age. Previous COVID-19 infection was associated with any daily cough and especially with acute and subacute cough. Allergy was a shared risk factor in acute and subacute cough in the elderly, consistent with the result in younger adults [5]. However, due to small number of subjects with acute and subacute cough, the analyses about their risk factors may have suffered from lack of statistical power.

Bronchiectasis, often presenting with chronic productive cough, is an uncommon condition with its highest prevalence in the elderly [25, 26]. Also in this population, bronchiectasis was uncommon (0.7%), but it increased the risk of chronic cough by sixfold. Previously, in the Danish study of general adult population, bronchiectasis was also the strongest risk factor for chronic cough in an individual level [6].

Asthma, chronic rhinosinusitis, and GORD were also strong risk factors of chronic cough in the elderly, corroborating earlier findings [13, 27]. Previously, in a study of 30 elderly patients, this triad explained about 85% of cases with chronic cough [27]. However, as many as 10 risk factors of chronic cough were now identified, suggesting that a wide range of background disorders need to be considered when evaluating chronic cough of an elderly person.

Somatic symptom score was used in this study as an indicator of the subject’s symptom burden, which may reflect the number of comorbidities, but also the individual tendency to recognise and report somatic symptoms. Thus, somatic symptom score allows suggestive consideration of somatisation in the risk factor analyses, which is relevant when investigating self-reported symptoms.

In Finnish working age adults in 2017, family history of chronic cough was recognised as a risk factor for all cough subtypes [5]. Since then, family history of chronic cough was reported as a potential predictor of chronic-persistent cough in Korea [28]. In the present study, family history of chronic cough was associated with subacute and chronic cough in Finnish elderly. The mechanism of the association is so far unknown.

The most common age in patients attending specialised chronic cough clinics is 60–69 years [29]. However, in the present community-based population, the prevalence of chronic cough increased up to over 80 years and by 20% per decade. Thus, it seems that very elderly chronic cough patients are underrepresented in specialised cough clinics. In community-based studies, the chronic cough prevalence trends have not been completely consistent, and decade-specific figures are often not presented in the most elderly. However, studies of older general populations suggest that the prevalence of chronic cough increases at least up to 80 years [6, 8, 9, 12].

Corroborating earlier studies in older general populations [8, 9], COPD was associated with chronic cough in this elderly population, although smoking status was not. Current smokers were underrepresented in the present study compared to general elderly population in Finland (1.7% *vs.* 7%) [30], which may have obscured the association of smoking with chronic cough.

OSA is recognised as a cause of chronic cough [31, 32]. In this study, OSA was associated with all cough subtypes. The STOP-questionnaire has a high sensitivity but moderate specificity in detecting OSA [18, 19]. Thus, overrepresentation of OSA in the risk factor analyses is possible.

Gender, BMI, and medications for arterial hypertension or diabetes were not associated with chronic cough in this study. However, the use of angiotensin-converting enzyme inhibitor or sitagliptin were not separately investigated. The associations of GORD and OSA with chronic cough were independent from BMI.

Here are the limitations of this study. The response rate was low, which is typical for email-based surveys. The generalisability of the results may suffer from the preponderance of females in the target population (63.5%) compared to general Finnish elderly (55.6%) [33]. However, the sex and age distribution of the respondents (mean age 72.2 years, 66.4% female) resembled those of the target population (mean age 72.7 years, 63.5% female). Causality cannot be confirmed in a cross-sectional study, and a survey study is vulnerable for selection bias. Furthermore, an email survey excludes people with severe decline in daily functioning. There are also several strengths to this study. It offered information about the point prevalence and risk factors of cough subtypes specifically in an elderly community-based population, which, to our knowledge, has not been done before. The use of point prevalence instead of period prevalence eliminates the problem of recall bias. Also, this was a twin study to our previous study, which was conducted in the same early spring season and using virtually the same questionnaire and definitions (except for OSA). This allows easy comparison of the results in elderly and non-elderly Finnish populations. Another strength of the study was the use of validated symptom questionnaires to define important background disorders of cough. By this way, even undiagnosed but symptomatic background disorders were considered in the risk factor analyses.

In conclusion, acute and subacute coughs, as well as previous COVID-19 infection, were uncommon in this elderly population during the COVID-19 pandemic in spring 2021. Consequently, the prevalence of any cough was lower than that previously found in younger adults. In theory, these results could be due to the use of personal protective and social measures and the resulting decrease in ARI incidence. The prevalence of chronic cough was higher in Finnish elderly than in younger Finnish adults and it increased up to the 8^th^ decade. This may result from the increase in the prevalence of various cough background disorders with ageing. When evaluating chronic cough of an elderly person, several background disorders should be considered, including asthma, chronic rhinosinusitis, GORD, COPD, OSA, and bronchiectasis.

## Supplementary Information

Below is the link to the electronic supplementary material.

Supplementary file1 (PDF 223 kb)
