# Supplementary material for: Cough in the Elderly During the COVID-19 Pandemic
Source: Lung. 2022 Mar 17;200(2):161–8. doi: 10.1007/s00408-022-00525-2 (PMC8927524; doi:10.1007/s00408-022-00525-2)
Supplement: Supplementary file 1 — Supplementary file1 (PDF 223 kb) [file 408_2022_525_MOESM1_ESM.pdf]

Supplementary file for the article "Cough in the Elderly During the COVID-19 Pandemic" in Lung

Authors

1. Johanna Tuulikki Kaulamo[2,3], 2. Anne Marika Lätti[1], 3. Heikki Olavi Koskela[1,2]

Affiliations of the authors

1. Unit for Medicine and Clinical Research, Pulmonary Division, Kuopio University Hospital, Kuopio, Finland
2. School of Medicine, Institute of Clinical Sciences, Faculty of Health Sciences, University of Eastern Finland, Finland
3. Mehiläinen Terveyspalvelut Oy, Health care services for prisoners, Kuopio, Finland

Corresponding author

Johanna Kaulamo, School of Medicine, Institute of Clinical Sciences, Faculty of Health Sciences, University of Eastern Finland, Yliopistonranta 1, 70210 Kuopio, Finland. Email: kaulamo@uef.fi.

COUGH IN SAVO AND CENTRAL FINLAND

THE QUESTIONNAIRE

GENERAL QUESTIONS

1. Gender

1 male
0 female

2. Year of birth___________

3. Place of birth_____________________________

4. Residence at the moment_________________________

5. Marital status

1 married/ in a registered relationship/ common-law marriage
2 unmarried
3 separated or divorced
4 widow/widower
5 can not define

6. How many years of full-time education have you had?
Please include both primary and secondary schooling

______years

7. In addition to yourself, how many people live in your household?

________individuals

8. At present, do you have any pets (also include farm animals if you live on a farm)?

0 no (please proceed to question 10)
1 yes

9. Do you have any of the following pets?

	no	yes
Dog	0	1
Cat	0	1
Rodent (mouse, hamster, guinea pig etc.)	0	1
Some other furry animal	0	1
Farm animals (cows, pigs etc.)	0	1
Some other animal	0	1

10. During the past 12 months has moisture damage been identified in your home?

0 no
1 yes

11. How large is your household's yearly income (before tax and other deductions) adding the income of all of its members together?

1 less than 15 000 e
2 15 000- 40 000 e
3 40 000 – 70 000 e
4. 70 000 – 120 000 e
5. More than 120 000 e

12. What was your most recent employment status?

1 employer, entrepreneur
2 farmer, farmer's wife
3 senior white collar worker
4 lower white collar worker
5 skilled blue collar worker (vocational college degree)
6 unskilled blue collar worker
7 I have not been employed outside the home

YOUR USE OF HEALTH SERVICES AND YOUR HEALTH CONDITION

13. How many times in the past year (12 months) have you visited a doctor for any symptom whatsoever?
If you have not visited a doctor at all in the past year, please write 0

_times

.
14. During the past year (12 months) have you had any of the following illnesses either diagnosed or treated by a doctor?

yes
asthma 1
chronic rhinitis……………………………….................................... 1
allergy (foods, pollen or to animals)……………...............................1
chronic obstructive pulmonary disease (= COPD)............................. 1
bronchiectasis…………………………………………………………1
fibrosing lung disease………………………………………………...1
sarcoidosis……………………………………………………………1
tuberculosis in the lungs..……………………………………………1
gastric distress (gastritis, gastric ulcer).……………………………...... 1
esophageal reflux disease (heartburn, gastroesophageal reflux)……..1
sleep apnea…………………………………………………………..…1
Parkinson's disease……………………………………………………1
depression 1
other mental health problems 1
rheumatoid arthritis……. 1
other connective tissue disorders (Sjögren's syndrome etc.)………....1
hypothyreosis……...……………………………………………………..1
arterial hypertension 1

elevated blood cholesterol 1
diabetes...1
myocardial infarction…………..1
coronary artery disease, angina pectoris …………………….......... 1
cancer 1
spinal disc problems, other back disease……………………………...1
prostate hyperplasia…………………………………………………1

15. Have you had any of the following symptoms or illnesses in the past month?

yes
chest pain on exertion 1
aching joints 1
back problems, back pain 1
toothache 1
swollen feet 1
varicose veins 1
eczema 1
headache 1
insomnia 1
depressive symptoms...1
other mental health problems 1
constipation 1
other gut problems (flatulence, diarrhoea) 1
sciatica (back pain that radiates to the leg) 1
urination disorders…………………………………………………1

16. Have you used any of the following drugs in the past month?

yes
cough medicines 1
drugs for asthma…………………………………………………..1
drugs for rhinitis……………………...1
drugs to treat allergies (for example, antihistamines).……………1
antacids (drugs to treat heartburn)………………………………..1
antihypertensives (drugs to treat high blood pressure)...................1
cholesterol lowering drugs...1
thyroid hormone (Thyroxine)……………………………………..1
insulin 1
drugs to treat diabetes 1
analgesics (pain medicines) 1
contraceptives............................. ………………………………1
tranquilizers .... ……………………………………..1
sedatives (sleeping pills) 1
antidepressants 1
vitamins or mineral supplements 1
hormone drugs to treat menopausal
or post-menopausal symptoms (for women) 1
potency medicines (for men) 1

17. Are you usually able to do the following physical actions?

	no	yes
walking for about half a kilometer without stopping to rest	0.......	1
running for a short distance (about 100 meters)...	0.......	1
running for a longer distance (more than half a kilometer)..........................	0……	1

18. At present, do you feel that your state of health is in general:

1 good
2 quite good
3 about average
4 quite bad
5 bad

19. How tall are you?

_______ cm

20. How much do you weigh? (wearing light clothing)

_______ kg

EXERCISE

21. How often do you undertake either leisure-time physical activity or the equivalent while commuting to work that lasts for at least half an hour and which makes you at least slightly out of breath or sweaty?

1 every day
2 4–6 times a week
3 3 times a week
4 2 times a week
5 once a week
6 2–3 times a month
7 a few times in a year or even less
8 I have a handicap or illness that makes it impossible for me to exercise

22. How would you rate your present physical condition?

1 very good
2 quite good
3 satisfactory
4 quite bad
5 very bad
6 I do not know

SMOKING HABITS

23. Have you ever smoked at any time in your life?

0 no (proceed directly to question 29)
1 yes

24. During your life have you smoked at least 100 times?
(cigarettes, cigars or pipes)
0 no
1 yes

25. Have you ever smoked on a daily basis for at least one year? For how many years altogether?

0 I have never smoked on a daily basis
1 I have smoked on a daily basis for ____ years

26. Are you currently a smoker?
(cigarettes, cigars, pipes or electronic cigarrettes)

1 yes, on a daily basis
2 occasionally
3 I am no longer a smoker

27. When did you last smoke?
If you are a regular smoker, please mark 1

1 yesterday or today
2 between 2 days and a month ago
3 between one and six months ago
4 between six months and a year ago
5 between one to five years ago
6 between five to ten years ago
7 more than 10 years ago

28. On average, how much do you smoke now on a daily basis or did you smoke before you quit smoking?
Please answer every point. Put 0, if you have never smoked that type of tobacco product at all

About ______ manufactured cigarettes every day
About _______self-rolled cigarettes every day
About______ pipefuls every day
About______ cigars every day
About______doses of electronic cigarettes every day

29. Is there anyone who currently smokes either inside your home or in your workplace?
0 no
1 yes

ALCOHOL CONSUMPTION

30. In the past year (12 months) have you ever drank any alcoholic beverages (e.g. beer, wine, cider or strong alcoholic drinks)?

0 I have not consumed any (please proceed to question 32)
1 yes

33. How many glasses (counted as usual restaurant-sized glasses) or bottles of the following alcoholic beverages have you drank in the past week: if you have not drank any of the particular type of beverage, please mark 0 in that line

About ____bottles (1/3 l) of medium strength or strong beer
About ____bottles (1/3 l) of alcopops /long drinks
About ____glasses of strong alcohol (restaurant sized glasses)
About____glasses of wine or alcohol of similar strength (alcohol content over 5%)
About ____glasses of cider or low-strength wine (alcohol content about 5%)

SYMPTOMS OF DISEASES OR ILLNESSES

32. In the past year (12 months), have you experienced wheezing or a whistling sound when you breathe
0 no (please proceed to question 39)
1 yes

33. Have you experienced a wheezing or whistling sound when you breathe at times other than when you are suffering from a flu or an upper respiratory tract infection?
0 no
1 yes

34. Have you experienced a shortness of breath at the same time when your breathing is wheezy or whistling?

0 no
1 yes

35. Has the wheezing or whistling sound when you are breathing disturbed your sleep in the past year (12 months)?
0 no
1 yes

36. In the past year (12 months) have you ever woken up due to an attack of a shortness of breath?
0 no
1 yes

37. In the past year (12 months) have you ever woken up due to a coughing fit?
0 no
1 yes

38. In the past year (12 months) have you experienced any of the following rhinitis-related symptoms?
You can mark ”yes” to more than one option

	no	yes
nasal discharge (anterior or posterior nasal drip)	0	1
nasal blockage	0	1
facial pain or pressure	0	1
reduction/loss of smell	0	1

39. In the past year (12 months) have you experienced any of the following rhinitis-related symptoms in a prolonged fashion, for at least three months?
You can mark ”yes” to more than one option

	no	yes
nasal discharge (anterior or posterior nasal drip)	0	1
nasal blockage	0	1
facial pain or pressure	0	1
reduction/loss of smell	0	1

40. Are you supersensitive to pain killing drugs (causing skin rash, swelling of your face, feelings of shortness of breath)?

0 no
1 yes

41. In the past 12 months have you suffered from heartburn and/or regurgitation?

0 no (please proceed to question 44)
1 yes

42. In the past 3 months have you suffered from heartburn and/or regurgitation?

0 no (please proceed to question 44)
1 yes

43. How often in the past 3 months have you suffered from heartburn and/or regurgitation?

1 less often than once a month
2 at least once a month
3 at least once a week
4 every day

44. Do you snore loudly (louder than talking or loud enough to be heard through closed doors)?

0 no
1 yes

45. Do you often feel tired, fatigued, or sleepy during daytime?

0 no
1 yes

46. Has anyone observed you stop breathing during your sleep?

0 no
1 yes

47. Have you had a covid-19 (coronavirus) infection?

0 no (please proceed to question 49)
1 yes, how many weeks ago did the symptoms start?______________________________

48. If you have had a covid-19 (coronavirus) infection, were you hospitalised for at least overnight?

0 no
1 yes

49. Have you been vaccinated against covid-19 (coronavirus) infection?

0 no

1 yes, how many weeks ago did you get the latest vaccination?______________________________

YOUR MOOD

How often in the past two weeks have you been troubled by the following problems?

50. Little interest or pleasure in doing things?

0 not at all
1 several days
2 more than half the days
3 nearly every day

51. Feeling down, depressed, or hopeless?

0 not at all
1 several days
2 more than half the days
3 nearly every day

QUESTIONS CONCENTRATING ON COUGH

52. Do you have any close family members (father, mother, sisters, brothers) who are now suffering or have suffered from prolonged cough which has lasted over two months? (include also family members who have already passed away)

0 no
1 yes

53. Have you suffered from a phlegmy cough on most days or nights for at least three months yearly?
0 no
1 yes

54. In past 12 months, have you had a cough?
choose only one option

0 not at all (please proceed to the last page)
1 yes

55. In the past 12 months, have you suffered from episodes of cough, which have lasted for at least one week and during which you have coughed daily?

0 No
1 Yes

56. How many such episodes you have had in the past 12 months? (If none, please write 0)

_____________ episodes

57. Do you think that there is some outside trigger or stimulus which makes you cough or worsens an existing cough?

0 no (please proceed to question 60)
1 yes

58. Which of the following triggers make you cough or worsens an existing cough?
You can select more than one option

	yes
Upper respiratory tract infection ("'flu")…………………………	1
Subfreezing air……………………………………………...	1
Physical exercise……………………………………………….	1
Automobile exhaust fumes……………………………………...	1
Poor indoor air quality………………………………………….	1
Proximity to animals…………………………………………….	1
Pollens…………………………………………………………..	1
Cigarette smoke………………………………………...…….	1
Strong scents (perfumes, deodorants etc.)…………………………	1
Strong paints or fumes……………………………………..….	1
Speaking………………………………………………………….	1
Eating or drinking (during or soon after it)……………………...	1
Laughing………………………………………………………....	1
Deep inspiration………………………………………………….	1
Something else……………………………………….....…...	1

59. If you chose the alternative 'something else', please define the trigger

____
____

60. In the past 12 months, have you used cough medicines?
(Both those purchased from a pharmacy and herbal remedies)

0 no
1 yes

61. How many times in the past year (12 months) have you visited the doctor because of your cough?
If you have not made any visits, then please mark 0

_______times

62. In the past two weeks, have you had a cough?

0 no
1 yes

The last part of this questionnaire is intended only for those people who have had a cough in the past two weeks i.e. those people who responded "yes" to question 62. Others can proceed to the last page of the questionnaire.

THE FOLLOWING QUESTIONS ARE ONLY TO BE ANSWERED BY THOSE WHO HAVE BEEN SUFFERING FROM COUGH IN THE PAST TWO WEEKS

63. Did you have symptoms of 'flu' when your current cough began (fever, throat ache, rhinitis, muscle pain or arthralgia, headache)?

0 No
1 Yes
2 Do not remember or can not define

64. How often has your current cough been bothering you?

1. Several times a day
2. Every day at least once a day
3. Four to six days in a week
4. Two or three times a week
5. At least once every week
6. Less than weekly

65. For how long have you been troubled by your current cough?

1. Less than one week
2. Longer than one week, but less than three weeks
3. More than three weeks, but less than two months
4. More than two months, but less than one year
5. More than one year, but less than five years
6. More than five years, but less than ten years
7. More than ten years

66. Have you considered being examined by a doctor because of your current cough?

0 no
1 yes

67. How many times have you visited a doctor because of your current cough?
Answer 0, if you have not visited a doctor due to your cough

_____times

The next questions are designed to assess the impact of cough on various aspects of your life. Read each question carefully and answer by choosing the response that best applies to you. Please answer all questions, as honestly as you can.

68. In the last 2 weeks, have you had chest or stomach pains as a result of your cough?

1. All of the time
2. Most of the time
3. A good bit of the time
4. Some of the time
5. A little of the time
6. Hardly any of the time
7. None of the time

69. In the last 2 weeks, have you been bothered by sputum (phlegm) production when you cough?

1. Every time

2. Most times
3. Several times
4. Some times
5. Occasionally
6. Rarely
7. Never

70. During the past two weeks, have you been tired because of your cough?

1. All of the time
2. Most of the time
3. A good bit of the time
4. Some of the time
5. A little of the time
6. Hardly any of the time
7. None of the time

71. In the last 2 weeks, have you felt in control of your cough?

1. None of the time
2. Hardly any of the time
3. A little of the time
4. Some of the time
5. A good bit of the time
6. Most of the time
7. All of the time

72. How often during the last 2 weeks have you felt embarrassed by your coughing?

1. All of the time
2. Most of the time
3. A good bit of the time
4. Some of the time
5. A little of the time
6. Hardly any of the time
7. None of the time

73. In the last 2 weeks, my cough has made me feel anxious.

1. All of the time
2. Most of the time
3. A good bit of the time
4. Some of the time
5. A little of the time
6. Hardly any of the time
7. None of the time

74. In the last 2 weeks, my cough has interfered with my job, or other daily tasks.

1. All of the time
2. Most of the time
3. A good bit of the time
4. Some of the time
5. A little of the time
6. Hardly any of the time

7. None of the time

75. In the last 2 weeks, I felt that my cough interfered with the overall enjoyment of my life.

1. All of the time
2. Most of the time
3. A good bit of the time
4. Some of the time
5. A little of the time
6. Hardly any of the time
7. None of the time

76. In the last 2 weeks, exposure to paints or fumes has made me cough.

1. All of the time
2. Most of the time
3. A good bit of the time
4. Some of the time
5. A little of the time
6. Hardly any of the time
7. None of the time

77. In the last 2 weeks, has your cough disturbed your sleep?

1. All of the time
2. Most of the time
3. A good bit of the time
4. Some of the time
5. A little of the time
6. Hardly any of the time
7. None of the time

78. In the last 2 weeks, how many times a day have you had coughing bouts?

1. All the time (continuously)
2. Most times of during the day
3. Several times during the day
4. Some times during the day
5. Occasionally through the day
6. Rarely
7. None

79. In the last 2 weeks, my cough has made me feel frustrated.

1. All of the time
2. Most of the time
3. A good bit of the time
4. Some of the time
5. A little of the time
6. Hardly any of the time
7. None of the time

80. In the last 2 weeks, my cough has made me feel fed up.

1. All of the time

2. Most of the time
3. A good bit of the time
4. Some of the time
5. A little of the time
6. Hardly any of the time
7. None of the time

81. In the last 2 weeks, have you suffered from a hoarse voice as a result of your cough?

1. All of the time
2. Most of the time
3. A good bit of the time
4. Some of the time
5. A little of the time
6. Hardly any of the time
7. None of the time

82. In the last 2 weeks, have you had a lot of energy?

1. None of the time
2. Hardly any of the time
3. A little of the time
4. Some of the time
5. A good bit of the time
6. Most of the time
7. All of the time

83. In the last 2 weeks, have you worried that your cough may indicate a serious illness?

1. All of the time
2. Most of the time
3. A good bit of the time
4. Some of the time
5. A little of the time
6. Hardly any of the time
7. None of the time

84. In the last 2 weeks, have you been concerned that other people think something is wrong with you, because of your cough?

1. All of the time
2. Most of the time
3. A good bit of the time
4. Some of the time
5. A little of the time
6. Hardly any of the time
7. None of the time

85. In the last 2 weeks, my cough interrupted conversation or telephone calls.

1. Every time
2. Most times
3. A good bit of the time
4. Some of the time
5. A little of the time

6. Hardly any of the time
7. None of the time

86. In the last 2 weeks, I feel that my cough has annoyed my partner, family or friends.

1. Every time I cough
2. Most times when I cough
3. Several times when I cough
4. Some times when I cough
5. Occasionally when I cough
6. Rarely
7. Never

CONSENT TO USE MY DATA, WHICH HAS BEEN CATHERED IN MEDICAL REGISTERS

You are able to participate in this questionnaire survey also without giving your personal identification data.

Can my data in medical registers (Statistics Finland, the Cancer Registry, and the National Institute for Health and Welfare) be utilised in association with the present study?

1 no. You can now submit the questionnaire without filling in the next sections – please click here___
2 yes. In that case, we ask you to fill in the next sections:

Cough in Savo and Central Finland
Unit for Medicine and Clinical Research, Pulmonary Division, Kuopio University Hospital, Kuopio,

I have been requested to participate in the above research project, which is intended to determine cough prevalence and to clarify factors that influence the prevalence and the consequences of cough in the population living in Eastern and Central Finland. I have read and understood the written information I have received about this research project. I have been given the opportunity to contact the researchers should I wish to receive further information. The information that I have received has given me a satisfactory understanding about this research project and about the way in which the data in the project will be gathered, handled and distributed. I was given a sufficient amount of time to consider whether or not I would participate in this project. I have been provided with sufficient information about my rights, the goals of this research project, how it will be implemented as well as the advantages and risks associated with this research project. I have not been coerced nor given any incentives to participate in this research project.

I understand that my participation is voluntary. I am aware that I can withdraw my permission to participate in this research project at any time and without giving any reason for my withdrawal. I am aware that my responses will be handled confidentially and not provided to any outside parties. I am aware that should I decide to interrupt or withdraw my permission to participate in this research project, any data which has been collected prior to my withdrawal/ refusal to continue can be included in the project’s research material.

Name of the participant ________________________________
Identity number of the participant_______________________
Address of the participant____
Telephone number of the paricipant_____________________
Date_______________________

You can now submit your responses by clicking here: ____
